# Correlation analyses between pre- and post-operative adverse events in gastric cancer patients receiving preoperative treatment and gastrectomy

**DOI:** 10.1186/s12885-016-2066-y

**Published:** 2016-01-19

**Authors:** Shuang-Xi Li, Sang Hyuk Seo, Yoon Young Choi, Masatoshi Nakagawa, Ji Yeong An, Hyoung-Il Kim, Jae-Ho Cheong, Woo Jin Hyung, Sung Hoon Noh

**Affiliations:** Department of Surgery, Yonsei University Health System, Yonsei University College of Medicine, 50 Yonsei-ro, Seodaemun-gu, 120-752 Seoul, South Korea; Key laboratory of Carcinogenesis and Translational Research (Ministry of Education), Department of Gastrointestinal Surgery, Peking University Cancer Hospital & Institute, Beijing, China; Department of Surgery, Inje University Busan Paik Hospital, Inje University College of Medicine, Busan, South Korea; Department of Gastric Surgery, Tokyo Medical and Dental University, Tokyo, Japan; Department of Surgery, Samsung Medical Center, Sungkyunkwan University School of Medicine, Seoul, South Korea

**Keywords:** Gastric cancer, Adverse event, Postoperative complication, Clavien-Dindo, CTCAE

## Abstract

**Backgrounds:**

Preoperative chemotherapy (PCT) and chemoradiotherapy (PCRT) showed promising results for gastric cancers. However, the influence of preoperative adverse events (AEs) on postoperative complications remains unknown. The aim of this study was to identify correlations between them.

**Methods:**

Clinical data and laboratory findings were retrieved retrospectively for 115 patients who underwent gastrectomy after PCT or PCRT between 2010 and 2013. Preoperative AEs and postoperative complications were classified according to the Common Terminology Criteria for Adverse Events (CTCAE) and Clavien-Dindo (CD) grading systems, respectively. Correlations between CTCAE grades and CD grades were analyzed, and clinical data and laboratory findings were compared among three groups classified according to CD grades: CD0, CD1/2, and CD3/4.

**Results:**

There were 61 (53.0 %) patients in the CD0 group, 44 (38.3 %) patients in the CD1/2 group, and 10 (8.7 %) patients in the CD3/4 group. The CTCAE grades did not correlate with the CD grades. Only estimated blood loss (*P* = 0.019) and transfusion rate (*P* < 0.001) differed among the three CD groups.

**Conclusion:**

There are no correlations between pre- and post-operative adverse events in the terms of severity grades in patients with advanced or metastatic gastric cancer who underwent gastrectomy after PCT or PCRT. Meticulous intraoperative manipulations should be emphasized.

## Background

Gastric cancer is the fifth most prevalent cancer worldwide, and more than 70 % of them occur in less developed countries [1], where they are often diagnosed at an advanced stage. D2 gastrectomy is regarded as the standard surgical treatment for locally advanced gastric cancer (AGC) based on randomized controlled trials [2, 3]. However, further treatment in addition to surgery is required to improve patient survival. The kind of additional treatment varies in different parts of the world: in East Asia, adjuvant chemotherapy after D2 gastrectomy is standard treatment for AGC [4, 5], whereas perioperative chemotherapy or postoperative chemoradiotherapy with gastrectomy is standard treatment in the West [6–8].

Recently, the use of preoperative chemotherapy (PCT) has gained increasing interest because of its possible advantages: 1) tumor down-staging increases the possibility of achieving complete surgical resection, 2) early application of chemotherapy may be effective in controlling micrometastases, 3) patients are more tolerable to chemotherapy before surgery, 4) the delivery of chemotherapeutic drugs will be enhanced because the blood supply to the tumor is intact, and 5) responses to chemotherapy are easily detected. PCT may even be useful for select patients with metastatic gastric cancer (MGC) prior to surgery [9, 10].

Despite these potential advantages, adverse events (AEs) due to PCT or preoperative chemoradiotherapy (PCRT) leading to deterioration of a patient’s physical condition could limit the ability to tolerate surgery. This is particularly relevant, as previously reported incidences of severe AEs ranged from 23.8 to 37.6 % [6, 7]. Thus, there are concerns that PCT or PCRT could increase postoperative morbidity [11]. Although postoperative morbidity after PCT or PCRT has been previously reported to be similar to that noted with surgery alone [6, 7, 12], to our knowledge, no previous studies have addressed the issue of whether preoperative AEs increase the likelihood or severity of postoperative complications. The aim of this study was to analyze the relationship between preoperative AEs and postoperative complications in patients who underwent gastrectomy for AGC or MGC after receiving PCT or PCRT.

## Methods

### Patients

**Fig. 1:**
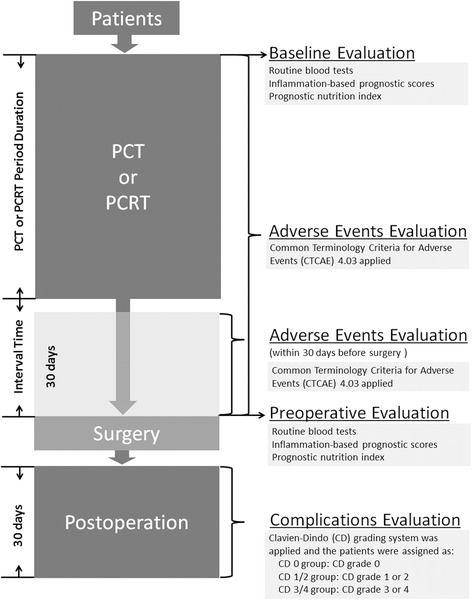
Study flow diagram. PCT, preoperative chemotherapy; PCRT, preoperative chemoradiotherapy

Patients were eligible for inclusion in this study if they had histologically-proven primary AGC or MGC, received initial PCT or PCRT, and underwent gastrectomy plus lymphadenectomy at Severance Hospital of Yonsei University between January 2010 and December 2013. The exclusion criteria included the presence of organ dysfunction before initial PCT or PCRT and surgery performed on an emergency basis. No comparative analysis was made to gastric cancer patient undergoing gastrectomy plus lymphadenectomy who did not receive initial PCT or PCRT. The clinical-pathological characteristics and laboratory investigations were retrieved from electronic medical records. The times for which data were collected are illustrated in Fig. 1. The study was performed in accordance with the Declaration of Helsinki, and was reviewed and approved by the Institutional Review Board of Severance Hospital, Yonsei University College of Medicine (4-2011-0991).

The regimens for PCT or PCRT varied from single-agent to triple-agent, they involved the use of fluorouracil, platinum, taxanes, and/or irinotecan. Intensity modulated radiation was performed using a dose of 40–45 Gy for PCRT. Since all patients received at least one type of fluorouracil-based regimen, we classified the chemotherapy regimens as platinum-containing, taxane-containing, containing both platinum and taxane, and others.

The operations included radical and palliative surgeries. As described in the Japanese Gastric Cancer Treatment Guidelines Version 3.0 [13], radical gastrectomy included resection of at least two-thirds of the stomach and peri-gastric lymphadenectomy with D2 extension. Palliative gastrectomy included resection of the entire gastric lesion or total gastrectomy plus lymphadenectomy with at least D1 extension.

### Baseline and preoperative evaluations

The time point definitions for baseline and preoperative evaluations were the most recent time before the initiation of PCT or PCRT and the surgery (after the final cycle) respectively. The laboratory investigations were collected for evaluations and included 3 categories of routine blood tests:

1.  Complete blood count: hemoglobin, white blood cell, neutrophil, lymphocyte, and platelet.
2.  Hepatorenal function: albumin, alanine transaminase, aspartate transaminase, alkaline phosphatase, and serum creatinine.
3.  Coagulation function: activated partial thromboplastin time, prothrombin, and international normalized ratio.

Additionally, we determined the neutrophil lymphocyte ratio (NLR), platelet lymphocyte ratio (PLR), and prognostic nutritional index (PNI), to evaluate the systemic inflammatory and nutritional status. Preoperatively, C-reactive protein was also recorded, and Glasgow Prognostic Score (GPS) and Prognostic Index (PI) were rated. All these values and factors were calculated and rated as described previously [14].

### Preoperative adverse events evaluation

Only the AEs based on objective laboratory investigations were classified according to the Common Terminology Criteria for Adverse Events (CTCAE) Version 4.03 [15], and they were anemia, leukopenia, neutropenia, febrile neutropenia, lymphocytopenia, thrombocytopenia, increased transaminase, and increased serum creatinine. The grade was determined by the highest CTCAE grade for each event, and the “Total AEs” grade referred to the highest CTCAE grade for all AEs in the study. Furthermore, AEs within 30 days before surgery were evaluated in an attempt to identify correlations between abnormal physical conditions in the month prior to surgery and postoperative complications.

### Postoperative complications evaluation

Postoperative complications within 30 days after surgery were classified according to the Clavien-Dindo (CD) grading system [16]. In our study, pulmonary-related complications included atelectasis, pleural effusion, or pneumonia, and infection-related complications consisted of gastrointestinal (GI) tract leakage, abdominal abscess, wound infection, pneumonia, or pyelonephritis. Patients with a temperature >38.5° centigrade and in whom other complications were subsequently excluded were diagnosed with an “unexplained fever”, which was classified as a grade 1 complication. The patients were assigned to one of the following three groups based on the severity of postoperative complications: no complications (CD0 group); grade 1 or 2 complications (CD1/2 group); and grade 3 or 4 complications (CD3/4 group).

### Statistical analyses

Nominal data are presented as number (percentage), scale data are presented as median (range), and AEs and complications are presented as number (incidence). Differences in nominal data and AEs among groups were detected by Chi-square tests or Fisher’s exact tests and differences in scale data among groups were detected by Kruskal-Wallis tests. Correlation analyses between CD grades and ordinal data, including CTCAE grades, GPS, and PI, were performed by Spearman’s correlation tests. A *P* value less than 0.05 (two-sided) was considered statistically significant. All analyses were performed with SPSS® version 22.0 (SPSS Inc., Chicago, IL, USA).

## Results

### Clinical-pathological characteristics

A total of 115 patients were included in this study. There were 74 males (64.3 %) and 41 females (35.7 %). The majority of patients received PCT, whereas only 23 patients (20.0 %) received PCRT. The postoperative stay differed among the three groups (*P* < 0.001). PCT or PCRT consisted of a median of 5.0 (1.0–21.0) cycles, and there were no significant differences among groups regarding other treatment-related parameters (Table 1). The operations primarily involved open surgery, total gastrectomy, radical resection, and D2 lymphadenectomy. Only estimated blood loss (EBL) (*P* = 0.017) and intra/postoperative transfusion rate (*P <* 0.001) were significantly different among groups (Table 2).

**Table 1:** Clinical characteristics and preoperative treatments^a^

	CD0 group (*n* = 61)	CD1/2 group (*n* = 44)	CD3/4 group (*n* = 10)	Total (*n* = 115)	*P* Value
Age^b^ (year)	56.0 (27.0–78.0)	56.0 (26.0–76.0)	58.5 (41.0–75.0)	56.0 (26.0–78.0)	0.467
Gender					0.452
Male	36 (59.0)	31 (70.5)	7 (70.0)	74 (64.3)	
Female	25 (41.0)	13 (29.5)	3 (30.0)	41 (35.7)	
BMI before surgery^b^ (kg/m^2^)	21.9 (17.3–30.7)	23.1 (17.1–32.4)	21.1 (16.9–29.0)	22.3 (16.9–32.4)	0.272
Tumor location					0.109
Proximal one-third	12 (19.7)	21 (47.7)	2 (20.0)	35 (30.4)	
Middle one-third	18 (29.5)	8 (18.2)	3 (30.0)	29 (25.2)	
Distal one-third	23 (37.7)	12 (27.3)	4 (40.0)	39 (33.9)	
Whole	8 (13.1)	3 (6.8)	1 (10.0)	12 (10.4)	
Preoperative treatment					0.881
PCT	50 (82.0)	34 (77.3)	8 (80.0)	92 (80.0)	
PCRT	11 (18.0)	10 (22.7)	2 (20.0)	23 (20.0)	
Cycles^b^	5.0 (2.0–20.0)	7.0 (2.0–19.0)	7.5 (2.0–21.0)	5.0 (2.0–21.0)	0.082
Duration^b^ (month)	3.1 (1.2–15.6)	4.5 (1.2–18.0)	5.4 (1.2–15.2)	3.6 (1.2–18.0)	0.061
Interval time^b^ (month)	1.1 (0.4–5.7)	1.0 (0.4–4.5)	1.2 (0.5–2.4)	1.1 (0.4–5.7)	0.167
Chemotherapy regimen					0.922
Platinum-containing	38 (62.3)	25 (56.8)	8 (80.0)	71 (61.7)	
Taxane-containing	7 (11.5)	6 (13.6)	1 (10.0)	14 (12.2)	
Containing both platinum and taxane	14 (23.0)	11 (25.0)	1 (10.0)	26 (22.6)	
Others	2 (3.3)	2 (4.5)	0 (0.0)	4 (3.5)	
Postoperative hospital stay^b^ (day)	8.0 (5.0–12.0)	10.0 (7.0–27.0)	19.0 (11.0–29.0)	9.0 (5.0–29.0)	<0.001

*BMI* body mass index, *PCRT* preoperative chemoradiotherapy, *PCT*, preoperative chemotherapy

^a^Data are presented as number (percentage) unless indicated otherwise

^b^Data are presented as median (range)

**Table 2:** Surgical and pathological characteristics ^a^

	CD0 group (*n* = 61)	CD1/2 group (*n* = 44)	CD3/4 group (*n* = 10)	Total (*n* = 115)	*P* Value
Surgical type					0.672
Open	55 (90.2)	42 (95.5)	9 (90.0)	106 (92.2)	
MIS	6 (9.8)	2 (4.5)	1 (10.0)	9 (7.8)	
Gastrectomy					0.219
Subtotal	31 (50.8)	15 (34.1)	5 (50.0)	51 (44.3)	
Total	30 (49.2)	29 (65.9)	5 (50.0)	64 (55.7)	
Surgical radicality					0.802
Radical	37 (60.7)	29 (65.9)	7 (70.0)	73 (63.5)	
Palliative	24 (39.3)	15 (34.1)	3 (30.0)	42 (36.5)	
Lymphadenectomy					0.504
D2	47 (77.0)	31 (70.5)	8 (80.0)	86 (74.8)	
D1 plus	13 (21.3)	10 (22.7)	1 (10.0)	24 (20.9)	
D1	1 (1.6)	3 (6.8)	1 (10.0)	5 (4.3)	
Combined resection					0.592
Yes	12 (19.7)	7 (15.9)	3 (30.0)	22 (19.1)	
No	49 (80.3)	37 (84.1)	7 (70.0)	93 (80.9)	
EBL^b^ (mL)	95 (28–300)	126 (30–609)	269 (50–640)	100 (28–640)	0.017
Transfusion					<0.001
Yes	0 (0.0)	17 (38.6)	1 (10.0)	18 (15.7)	
No	61 (100.0)	27 (61.4)	9 (90.0)	97 (84.3)	
Borrmann type					0.524
I	6 (9.8)	2 (4.5)	2 (20.0)	10 (8.7)	
II	12 (19.7)	10 (22.7)	1 (10.0)	23 (20.0)	
III	32 (52.5)	20 (45.5)	6 (60.0)	58 (50.4)	
IV	11 (18.0)	12 (27.3)	1 (10.0)	24 (20.9)	
Lauren type					0.569
Intestinal	30 (49.2)	23 (52.3)	6 (60.0)	59 (51.3)	
Diffuse	26 (42.6)	18 (40.9)	2 (20.0)	46 (40.0)	
Mixed	5 (8.2)	3 (6.8)	2 (20.0)	10 (8.7)	
TNM Stage					0.846
yp 0	4 (6.6)	1 (2.3)	1 (10.0)	6 (5.2)	
yp Ia-Ib	14 (23.0)	7 (15.9)	1 (10.0)	22 (19.1)	
yp IIa-IIb	6 (9.8)	6 (13.6)	2 (20.0)	14 (12.2)	
yp IIIa-IIIc	15 (24.6)	15 (34.1)	3 (30.0)	33 (28.7)	
yp IV	22 (36.1)	15 (34.1)	3 (30.0)	40 (34.8)	

EBL, Estimated blood loss; MIS, Minimal invasive surgery

^a^Data are presented as number (percentage) unless indicated otherwise

^b^Data are presented as median (range)

### Incidence and type of preoperative adverse events

Overall, the incidence rates for AEs during PCT or PCRT were as follows (in descending order): anemia (89.6 %, 103/115), lymphocytopenia (81.7 %, 94/115), neutropenia (80 %, 92/115), leukopenia (67.8 %, 78/115), increased transaminase (40.9 %, 47/115), thrombocytopenia (32.2 %, 37/115), febrile neutropenia (19.1 %, 22/115), and increased serum creatinine (6.1 %, 7/115). The total AEs incidence was 99.1 % (114/115). For serious AEs classified as CTCAE grade 3/4, the incidence rates were as follows (in descending order): neutropenia (47.0 %, 54/115), leukopenia (28.7 %, 33/115), anemia (19.1 %, 22/115), lymphocytopenia (16.5, 19/115), febrile neutropenia (12.2 %, 14/115), and thrombocytopenia (4.3 %, 5/115). The total serious AEs incidence was 61.7 % (71/115). The distributions of AE severity (ratios of grade 0, grade 1/2, and grade 3/4) were comparable among groups (Table 3).

**Table 3:** Incidence of AEs during PCT or PCRT according to CTCAE and Clavien-Dindo grades^a^

	CD0 group (*n* = 61)	CD1/2 group (*n* = 44)	CD3/4 group (*n* = 10)	Total (*n* = 115)	*P* Value
Anemia					0.099
Grade 0	6 (5.2)	6 (5.2)	0 (0.0)	12 (10.4)	
Grade 1/2	48 (41.7)	27 (23.5)	6 (5.2)	81 (70.4)	
Grade 3/4	7 (6.1)	11 (9.6)	4 (3.5)	22 (19.1)	
Leukopenia					0.326
Grade 0	24 (20.9)	9 (7.8)	4 (3.5)	37 (32.2)	
Grade 1/2	21 (18.3)	21 (18.3)	3 (2.6)	45 (39.1)	
Grade 3/4	16 (13.9)	14 (12.2)	3 (2.6)	33 (28.7)	
Neutropenia					0.244
Grade 0	16 (13.9)	4 (3.5)	3 (2.6)	23 (20.0)	
Grade 1/2	19 (16.5)	16 (13.9)	3 (2.6)	38 (33.0)	
Grade 3/4	26 (22.6)	24 (20.9)	4 (3.5)	54 (47.0)	
Febrile neutropenia					0.829
Grade 0	49 (42.6)	35 (30.4)	9 (7.8)	93 (80.9)	
Grade 3/4	12 (10.4)	9 (7.8)	1 (0.9)	22 (19.1)	
Lymphocytopenia					0.748
Grade 0	10 (8.7)	9 (7.8)	2 (1.7)	21 (18.3)	
Grade 1/2	41 (35.7)	29 (25.2)	5 (4.3)	75 (65.2)	
Grade 3/4	10 (8.7)	6 (5.2)	3 (2.6)	19 (16.5)	
Thrombocytopenia					0.798
Grade 0	39 (33.9)	26 (22.6)	8 (7.0)	73 (63.5)	
Grade 1/2	19 (16.5)	16 (13.9)	2 (1.7)	37 (32.2)	
Grade 3/4	3 (2.6)	2 (1.7)	0 (0.0)	5 (4.3)	
Increased ALT/AST					0.718
Grade 0	34 (29.6)	28 (24.3)	6 (5.2)	68 (59.1)	
Grade 1/2	27 (23.5)	16 (13.9)	4 (3.5)	47 (40.9)	
Increased Scr					1.000
Grade 0	58 (50.4)	41 (35.7)	9 (7.8)	108 (93.9)	
Grade 1/2	3 (2.6)	3 (2.6)	1 (0.9)	7 (6.1)	
Total AEs					0.138
Grade 0	0 (0.0)	1 (0.9)	0 (0.0)	1 (0.9)	
Grade 1/2	29 (25.2)	11 (9.6)	3 (2.6)	43 (37.4)	
Grade 3/4	32 (27.8)	32 (27.8)	7 (6.1)	71 (61.7)	

*AEs* adverse events, *ALT* alanine transaminase, *AST* aspartate transaminase, *CTCAE* common terminology criteria for adverse events, *PCRT* preoperative chemoradiotherapy, *PCT* preoperative chemotherapy, *Scr* serum creatinine

^a^Data are presented as number (incidence)

For AEs within 30 days before surgery, the AE incidence rates were as follows (in descending order): anemia (67.0 %, 77/115), lymphocytopenia (51.3 %, 59/115), neutropenia (37.4 %, 43/115), leukopenia (34.8 %, 40/115), and thrombocytopenia (23.5 %, 27/115). The total AEs incidence was 80.0 % (92/115), including a 16.5 % (19/115) incidence of grade 3/4 AEs. The distributions of AE severity (ratios of grade 0, grade 1/2, and grade 3/4) did not differ among groups (Table 4).

**Table 4:** Incidence of AEs within 30 days before surgery according to CTCAE and Clavien-Dindo grades^a^

	CD0 group (*n* = 61)	CD1/2 group (*n* = 44)	CD3/4 group (*n* = 10)	Total (*n* = 115)	*P* Value
Anemia					0.724
Grade 0	23 (20.0)	13 (11.3)	2 (1.7)	38 (33.0)	
Grade 1/2	36 (31.3)	30 (26.1)	8 (7.0)	74 (64.3)	
Grade 3/4	2 (1.7)	1 (0.9)	0 (0.0)	3 (2.6)	
Leukopenia					0.196
Grade 0	38 (33.0)	27 (23.5)	10 (8.7)	75 (65.2)	
Grade 1/2	19 (16.5)	15 (13.0)	0 (0.0)	34 (29.6)	
Grade 3/4	4 (3.5)	2 (1.7)	0 (0.0)	6 (5.2)	
Neutropenia					0.740
Grade 0	38 (33.0)	26 (22.6)	8 (7.0)	72 (62.6)	
Grade 1/2	16 (13.9)	12 (10.4)	2 (1.7)	30 (26.1)	
Grade 3/4	7 (6.1)	6 (5.2)	0 (0.0)	13 (11.3)	
Lymphocytopenia					0.720
Grade 0	31 (27.0)	19 (16.5)	6 (5.2)	56 (48.7)	
Grade 1/2	27 (23.5)	24 (20.9)	4 (3.5)	55 (47.8)	
Grade 3/4	3 (2.6)	1 (0.9)	0 (0.0)	4 (3.5)	
Thrombocytopenia					0.577
Grade 0	45 (39.1)	34 (29.6)	9 (7.8)	88 (76.5)	
Grade 1/2	16 (13.9)	10 (8.7)	1 (0.9)	27 (23.5)	
Total AEs					0.421
Grade 0	14 (12.2)	8 (7.0)	1 (0.9)	23 (20.0)	
Grade 1/2	36 (31.3)	28 (24.3)	9 (7.8)	73 (63.5)	
Grade 3/4	11 (9.6)	8 (7.0)	0 (0.0)	19 (16.5)	

*AEs* adverse events, *CTCAE* common terminology criteria for adverse events

^a^Data are presented as number (incidence)

### Incidence and type of postoperative complications

There was no postoperative mortality, and the morbidity rate was 47.0 % (54/115). The most frequent complication was pulmonary-related complications, the most common minor complications (CD1/2) were pulmonary-related complications and transfusion, and the most common serious complication (CD3/4) was GI tract leakage/abscess (Table 5).

**Table 5:** Postoperative complications^a^

	CD1/2 group (*n* = 44)	CD3/4 group (*n* = 10)	Total^b^ (*n* = 115)
Non-surgery-related			
Pulmonary-related	17 (14.8)	3 (2.6)	20 (17.4)
Deep vein thrombus	1 (0.9)	0 (0.0)	1 (0.9)
Unexplained fever	7 (6.1)	0 (0.0)	7 (6.1)
Transfusion	17 (14.8)	1 (0.9)	18 (15.7)
Others^c^	3 (2.6)	1 (0.9)	4 (3.5)
Surgery-related			
Wound discharge/infection	5 (4.3)	2 (1.7)	7 (6.1)
Intestinal obstruction	3 (2.6)	1 (0.9)	4 (3.5)
Fluid collection	9 (7.8)	1 (0.9)	10 (8.7)
Gastrointestinal tract leakage/abscess	1 (0.9)	6 (5.2)	7 (6.1)
Complication type			
Not infection-related	26 (22.6)	3 (2.6)	29 (25.2)
Infection-related	12 (10.4)	7 (6.1)	19 (16.5)
Clavien-Dindo grade			
Grade 1	15 (13.0)	NA	15 (13.0)
Grade 2	29 (25.2)	NA	29 (25.2)
Grade 3	NA	8 (7.0)	8 (7.0)
Grade 4	NA	2 (1.7)	2 (1.7)

*NA* not applicable

^a^Data are presented as number (incidence)

^b^Included 61 patients in CD0 group

^c^Included pyelonephritis, hydronephrosis, re-admission for nutrition support, and renal dysfunction

### Correlation analyses of preoperative adverse events and postoperative complications

**Fig. 2:**
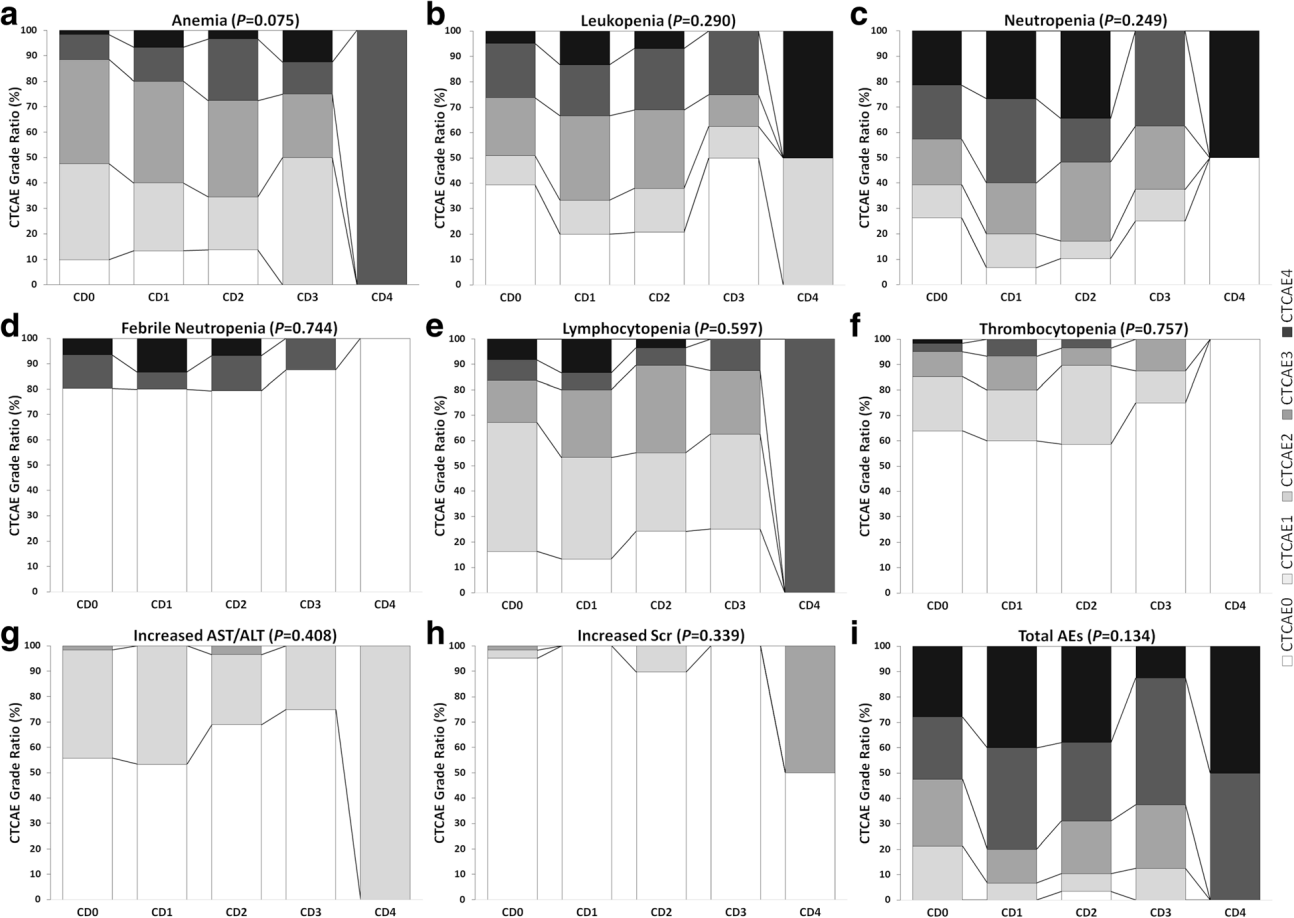
Stacked percentage bar charts for AEs during PCT and PCRT. AEs, adverse events; ALT, alanine transaminase; AST, aspartate transaminase; CTCAE, Common Terminology Criteria for Adverse Events; PCRT, preoperative chemoradiotherapy; PCT, preoperative chemotherapy; Scr serum creatinine. Correlation analysis was performed by Spearman’s correlation test. The charts from **a**-**i** refer to the stacked percentage for anemia, leukopenia, neutropenia, febrile neutropenia, lymphocytopenia, thrombocytopenia, increased AST/ALT, increased Scr, and total AEs respectively

**Fig. 3:**
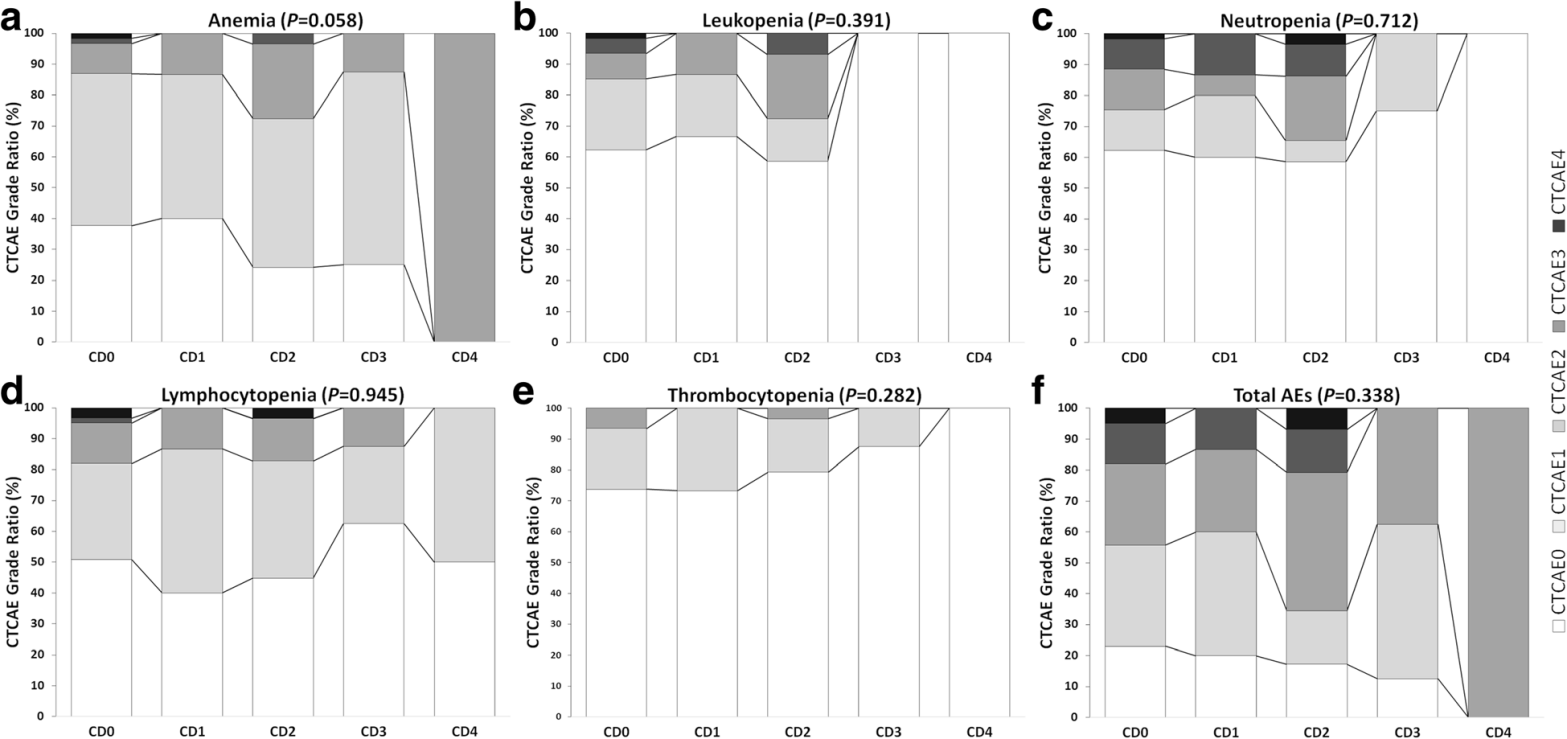
Stacked percentage bar charts for AEs within 30 Days before surgery. AEs, adverse events; CTCAE, Common Terminology Criteria for Adverse Events; PCRT, preoperative chemoradiotherapy; PCT, preoperative chemotherapy. Correlation analysis was performed by Spearman’s correlation test. The charts from **a**-**f** refer to the stacked percentage for anemia, leukopenia, neutropenia, lymphocytopenia, thrombocytopenia, and total AEs respectively

Correlation analyses were performed between the CTCAE grade of AEs during PCT or PRCT and the CD grade of postoperative complications using Spearman’s correlation tests. The results revealed no correlations between the two grading systems (Fig. 2). Similarly, no correlations were noted when comparing the CTCAE grades of AEs within 30 days before operation and the CD grade of postoperative complications (Fig. 3).

### Differences in laboratory tests, inflammation-based prognostic scores, and nutrition index

During baseline and preoperative evaluations, all laboratory test results were comparable among groups, with no statistically significant differences. Similarly, the NLR, PLR, and PNI at baseline and preoperatively failed to exhibit significant differences among groups. According to Spearman’s correlation tests, neither the preoperative GPS nor PI was correlated with the postoperative complications CD grade (Table 6).

**Table 6:** Differences in laboratory tests, inflammation-based prognostic scores, and nutrition index according to Clavien-Dindo grades^a^

	CD0 group (*n* = 61)	CD1/2 group (*n* = 44)	CD3/4 group (*n* = 10)	*P* Value
Baseline				
HB (g/L)	129 (66–155)	121 (64–170)	112 (72–159)	0.284
WBC (×10^9^/L)	5.9 (3.6–13.9)	6.4 (3.6–14.2)	8.1 (3.9–10.2)	0.109
NEU (×10^9^/L)	3.6 (2.2–10.9)	3.9 (2.0–11.7)	4.8 (1.4–7.7)	0.136
LYM (×10^9^/L)	1.7 (0.7–3.1)	1.8 (0.6–3.0)	2.0 (1.4–2.4)	0.238
PLT (×10^9^/L)	298.0 (96.0–639.0)	295.0 (141.0–599.0)	301.5 (166.0–438.0)	0.784
ALB (g/L)	42.0 (35.0–49.0)	41.0 (28.0–47.0)	39.0 (25.0–48.0)	0.631
ALT (U/L)	14.0 (5.0–75.0)	14.5 (5.0–58.0)	13.0 (7.0–59.0)	0.888
AST (U/L)	17.0 (10.0–56.0)	15.0 (8.0–73.0)	19.5 (10.0–37.0)	0.473
ALP (U/L)	56.0 (24.0–109.0)	59.5 (25.0–123.0)	71.5 (31.0–99.0)	0.137
Scr (mg/dL)	0.82 (0.48–1.20)	0.86 (0.53–1.28)	0.81 (0.68–1.59)	0.593
aPTT (second)	29.8 (23.0–37.2)	29.6 (24.9–38.6)	28.6 (24.9–33.1)	0.315
PT (second)	10.9 (9.3–12.8)	11.0 (9.0–13.1)	11.1 (9.5–12.7)	0.765
INR	0.96 (0.82–1.12)	0.97 (0.79–1.16)	0.98 (0.84–1.11)	0.883
PNI	50.2 (40.4–61.8)	50.6 (34.2–60.0)	49.7 (33.0–60.2)	0.954
NLR	2.5 (0.7–6.6)	2.2 (1.0–9.5)	2.4 (0.7–7.8)	0.805
PLR	181.9 (67.6–557.7)	179.1 (73.8–487.0)	171.1 (78.3–256.9)	0.683
Preoperative				
HB (g/L)	116 (86–152)	115 (76–160)	117 (91–125)	0.810
WBC (×10^9^/L)	4.7 (2.3–11.7)	4.7 (2.7–11.9)	5.8 (3.0–9.7)	0.598
NEU (×10^9^/L)	2.7 (0.7–8.8)	2.6 (1.2–9.1)	3.0 (1.5–7.8)	0.755
LYM (×10^9^/L)	1.5 (0.5–3.4)	1.6 (0.4–2.7)	1.3 (0.6–2.9)	0.835
PLT (×10^9^/L)	218.0 (73.0–398.0)	202.0 (91.0–583.0)	216.5 (167.0–276.0)	0.861
ALB (g/L)	40.0 (27.0–48.0)	39.5 (26.0–52.0)	39.0 (32.0–41.0)	0.541
ALT (U/L)	17.0 (6.0–72.0)	14.0 (6.0–52.0)	15.5 (11.0–43.0)	0.431
AST (U/L)	20.0 (12.0–75.0)	19.0 (11.0–42.0)	19.0 (12.0–42.0)	0.671
ALP (U/L)	61.5 (21.0–142.0)	59.5 (25.0–106.0)	63.5 (35.0–163.0)	0.887
Scr (mg/dL)	0.78 (0.43–1.39)	0.78 (0.38–1.55)	0.75 (0.63–1.34)	0.874
aPTT (second)	29.9 (24.2–41.5)	29.7 (20.7–37.5)	31.0 (26.1–34.6)	0.979
PT (second)	10.5 (9.4–13.1)	10.9 (9.4–13.4)	10.5 (9.6–11.7)	0.196
INR	0.93 (0.83–1.15)	0.97 (0.83–1.18)	0.94 (0.85–1.01)	0.147
CRP^b^ (mg/L)	1.1 (0.3–51.1)	1.3 (0.4–68.6)	1.8 (0.5–13.9)	0.502
PNI	46.8 (30.5–59.9)	46.0 (33.2–62.1)	45.4 (37.2–54.3)	0.801
NLR	1.7 (0.6–9.6)	1.9 (0.6–9.7)	2.0 (0.8–10.1)	0.811
PLR	134.6 (38.2–574.1)	123.4 (44.7–640.7)	148.3 (66.1–392.1)	0.714
GPS^b,c^				0.804
Score 0	43 (81.1)	30 (76.9)	7 (87.5)	
Score 1	8 (15.1)	6 (15.4)	0 (0.0)	
Score 2	2 (3.8)	3 (7.7)	1 (12.5)	
PI^b,c^				0.572
Score 0	49 (92.5)	35 (89.7)	7 (87.5)	
Score 1	4 (7.5)	4 (10.3)	1 (12.5)	

ALB, albumin; ALP, alkaline phosphatase; ALT, alanine transaminase; aPTT, activated partial thromboplastin time; AST, aspartate transaminase; CRP, C-reactive protein; GPS, Glasgow Prognostic Score; HB, hemoglobin; INR, international normalized ratio; LYM, lymphocyte count; NEU, neutrophil count; NLR, neutrophil–lymphocyte ratio; PI, Prognostic Index; PLR, platelet–lymphocyte ratio; PLT, platelet count; PNI, prognostic nutritional index; PT, prothrombin; Scr, serum creatinine; WBC, white blood cell count

^a^Data are presented as median (range) unless indicated otherwise

^b^Data retrieved from 100 patients, including 53 patients in CD0 group, 39 patients in CD1/2 group, and 8 patients in CD3/4 group

^c^Data are presented as number (percentage)

## Discussion

In this retrospective study, we focused on the relationship between preoperative AEs and postoperative complications in patients with AGC or MGC who received PCT or PCRT. The incidence of both total AEs and total serious AEs were extremely high compared to previous studies [6, 7, 17]. The 5.0 median cycles of PCT or PCRT in our study may have contributed to this discrepancy, as it is reasonable to expect a higher incidence of AEs in our population who received extensive preoperative treatment. Even in the time period very close to surgery (within 30 days before surgery), we observed a relatively high incidence of total AEs. The AEs associated with PCT or PCRT would definitely negatively affect a patient’s physical condition, which may subsequently influence the postoperative course; thus, concerns have arisen about the safety of performing gastrectomy and lymphadenectomy for such vulnerable patients.

A correlation between poor physical status and postoperative morbidity was previously reported by Jung et al. [18], who indicated that patients with preoperative anemia had a higher morbidity rate after radical gastrectomy than patients without. By contrast, the CTCAE grades for AEs in our study did not correlate with postoperative CD grades. A similar result was reported by Reim et al. [19], who noted that preoperative leukopenia due to neoadjuvant chemotherapy had no significant effect on postoperative complications after total gastrectomy plus D2 lymphadenectomy. However, because there were many discrepancies in patient selection, evaluation criteria, surgical procedures, and diagnostic standards among the previous studies, it was difficult to make definitive conclusions from these studies. The results of our current study can reduce concerns regarding the safety of surgery after PCT or PCRT, but they cannot eliminate them. A well-designed clinical trial is required to provide firm conclusions regarding this issue.

In previous studies, postoperative morbidity rates after gastrectomy plus lymphadenectomy varied from 25.7 to 45.7 % in patients who underwent PCT or PCRT [6, 7, 11, 12]. The morbidity rate of 47.0 % in our study is relatively high but still comparable to the rate noted in a previous report from our institute [11]. In the present study, a high percentage of patients had stage IV cancer and underwent total gastrectomy and combined resection, which suggests that their surgery was more complicated. Postoperative morbidity may have been increased for this reason. It should be noted that the most common minor (CD1/2) complication in our study was pulmonary-related complications, a type of non-surgery-related complications. It is quite possible that these complications were related to a poor physical status of patients after PCT or PCRT. Additionally, the relatively low incidence of serious (CD3/4) complications (8.7 %) highlights the advantage of performing such complex surgery in a high-volume center.

Higher intraoperative blood loss and operative transfusion rates were previously reported to correlate with postoperative morbidity after gastrectomy and lymphadenectomy [11, 20]. In terms of CD grades, we found significant differences among groups for EBL and transfusion rates. Patients with greater CD grades had a greater EBL. In the CD grading system, transfusion is classified as a grade 2 morbidity, which was why the transfusion rate was highest in our CD1/2 group. Generally, a higher blood loss reflects increased surgical difficulty and correlates with a higher transfusion rate. For the same level of surgical complexity, gastrectomy could be more difficult in patients who received PCT or PCRT because of peri-tumoral fibrosis, edema, or a bulky tumor mass. These factors are known to increase the need for surgical technique and experience. Independent of other factors related to postoperative morbidity, the surgical technique and quality are definitely crucial determinants of outcome. Meticulous manipulations during surgery should reduce trauma, operative blood loss, and ultimately morbidity.

In recent years, certain factors derived from routine laboratory investigations have been reported to manifest prognostic values in predicting the outcomes for patients with AGC and MGC. These factors include white blood cell and neutrophil counts, NLR, PLR, GPS, and PNI [14, 21, 22]. Importantly, Jiang et al. [23] reported that the severity of postoperative complication could influence the survival time for patients with gastric cancer, thereby suggesting the possibility of a direct link between the severity of postoperative morbidity and long-term prognostic factors. However, in our study, all laboratory findings, inflammation-based prognostic scores, and PNI did not differ among groups. The discrepancy is quite possibly because of the PCT or PCRT, as the baseline parameters no longer represented the patient’s actual preoperative status, and the preoperative parameters could have been altered to varying degrees. Therefore, the above factors do not appear to be useful for predicting the postoperative morbidity for patients who received PCT or PCRT.

Limitations of this study include the relatively small number of patients and the natural drawbacks associated with all retrospective studies. There was substantial heterogeneity among patients and interventions. Firstly, patients with serious preoperative AEs tended to be under intensive clinical observations and may have received therapeutic interventions, which may have influenced postoperative morbidity. Secondly, the extent of surgery differed among patients. The surgical extent itself can influence postoperative morbidity, and should be well defined in a future study. Thirdly, the PCT and PCRT had different systemic and local effects, and each specific regimen also had unique toxicities. The variable treatments and regimens may have led to differing physiological alterations, even under the same AE grade. Fourthly, there were a number of patients who received preoperative treatment with PCT or PCRT but failed to subsequently proceed on to surgery. Data regarding the exact number of these patients nor the reasons why they did not subsequently proceed on to surgery was not included within the current study analysis. Therefore, had these individuals proceeded on to surgery after receiving preoperative treatment with PCT or PCRT, the observed degree and severity of postoperative complications that occurred in the overall study group could have been substantially different. However, there is no realistic way for us to make any such inferences into how preoperative AEs in this particular group of patients may have impacted upon the overall postoperative complications observed in the entire study group if this group of patients had theoretically proceeded on to surgery. Lastly, despite the limitations of our study, it is our belief that our use of both the CTCAE system for classifying AEs and the CD grading system for classifying postoperative complications allowed for minimizing any subjectivity to the analysis process of the data included within the current study.

## Conclusions

In summary, for patients with AGC and MGC who underwent gastrectomy and lymphadenectomy, this study provided supportive evidence favoring the use of PCT or PCRT. The CTCAE grades of preoperative AEs did not correlate with the CD grades of postoperative complications. The higher EBL and transfusion rate noted in patients with complications emphasize the importance of meticulous manipulations during surgery to reduce postoperative morbidity.

## Abbreviations

AEs: Adverse events
AGC: Advanced gastric cancer
BMI: Body mass index
CD: Clavien-Dindo
CTCAE: Common terminology criteria for adverse events
EBL: Estimated blood loss
GI: Gastrointestinal
GPS: Glasgow prognostic score
MGC: Metastatic gastric cancer
MIS: Minimal invasive surgery
NA: Not applicable
NLR: Neutrophil lymphocyte ratio
PCRT: Preoperative chemoradiotherapy
PCT: Preoperative chemotherapy
PI: Prognostic index
PLR: Platelet lymphocyte ratio
PNI: Prognostic nutritional index
